# The Sam domain of the lipid phosphatase Ship2 adopts a common model to interact with Arap3-Sam and EphA2-Sam

**DOI:** 10.1186/1472-6807-9-59

**Published:** 2009-09-18

**Authors:** Marilisa Leone, Jason Cellitti, Maurizio Pellecchia

**Affiliations:** 1 Burnham Institute for Medical Research, La Jolla, California, USA

## Abstract

**Background:**

Sterile alpha motif (Sam) domains are small protein modules that can be involved in homotypic or heterotypic associations and exhibit different functions. Previous studies have demonstrated that the Sam domain of the lipid phosphatase Ship2 can hetero-dimerize with the Sam domain of the PI3K effector protein Arap3.

**Results:**

Here, we determine the NMR solution structure of Arap3-Sam and implement a multidisciplinary approach consisting of NMR spectroscopy, ITC (Isothermal Titration Calorimetry), mutagenesis and molecular modeling studies to analyze the interaction between Ship2-Sam and Arap3-Sam. This work reveals that Arap3-Sam may associate with Ship2-Sam by adopting a binding mode common to other Sam domains. This binding mode is identical to what we have very recently observed for the association between Ship2-Sam and the Sam domain from the Ephrin A2 receptor.

**Conclusion:**

Our studies further clarify the structural features that are relevant for Sam-Sam interactions involving Ship2 and give additional hints that could be used for the identification of new molecules able to selectively inhibit Sam-Sam associations.

## Background

Arap3 is a protein involved in phospatidylinositol 3 kinase (PI3K) signaling pathways linked to regulation of the actin cytoskeleton, cell spreading and the formation of lamellipodia [1,2]. It works as a GTP-ase activator protein (GAP) for the small G-proteins Arf and Rho [2]. Previous studies have reported that Arap3 binds Ship2 (Src homology 2 domain-containing phosphoinositide-5-phosphatase 2) by forming a hetero-dimer via the sterile alpha motif domains (Sam) of both proteins [3]. The dissociation constant for this complex is about 100 nM as determined by previous Isothermal titration Calorimetry (ITC) studies [3].

Although the consequences of the interaction between the Sam domains of Arap3 (Arap3-Sam) and Ship2 (Ship2-Sam) are not completely clear, it has been speculated that the heterotypic Sam-Sam association may be used by Arap3 to link a negative regulator of PI3K signaling (i.e., Ship2) to the effector complex [3]. In fact, while Arap3 needs to bind phosphatidylinositol(3,4,5)P3 (PtIns(3,4,5)P3) with one of its pleckstrin homology (PH) domains to migrate to the plasma membrane and be activated [4]; Ship2, by converting PtIns(3,4,5)P3 to phosphatidylinositol(3,4)P2 (PtIns(3,4)P2), works as an inhibitor of PI3K regulated pathways [5,6].

Here, we report on structural and binding studies of Arap3-Sam and Ship2-Sam. First, we determined the NMR solution structure of the Sam domain of Arap3 and characterized its interaction site for Ship2-Sam. Furthermore, we also established the binding site of Ship2-Sam for Arap3-Sam. Based on our observations, we speculate that the Ship2-Sam/Arap3-Sam complex may adopt the ML (Mid Loop)/EH (End Helix) interaction model that is common to many Sam/Sam associations [7-9]. For example, we recently reported on a similar binding mode involving Ship2-Sam and the Sam domain from the Ephrin A2 receptor (EphA2-Sam) [10], a Sam-Sam association that is important to regulate receptor endocytosis [11]. In agreement with these data, our NMR displacement experiments confirmed that Arap3-Sam and EphA2-Sam compete for a common binding site on the surface of Ship2-Sam.

Our studies further clarify the structural features that are important for Sam-Sam interactions involving Ship2 and give additional hints useful for identifying new molecules able to selectively inhibit Sam-Sam associations.

## Results

### NMR Solution Structure of Arap3-Sam

The aggregation state of Arap3-Sam in solution was analyzed by means of analytical ultracentrifugation studies and backbone ^15^N R1 and R2 nuclear spin relaxation rates measurements.

Arap3-Sam has a rotational correlation time *τ*c estimated by the R2/R1 average value of 7.7 ± 0.7 ns at a protein concentration of 150 *μ*M which increases only slightly to 8.2 ± 0.4 ns at the concentration of 1.4 mM. The Arap3-Sam τc value is indeed similar to those reported for other Sam domains, including Ship2, which only weakly self-associate [10,12,13]. On the contrary, the *τ*c of Ship2-Sam bound to Arap3-Sam is 11 ± 1 ns, this higher value reflects the increase of the molecular weight caused by the Sam-Sam association and points towards the formation of a dimer [10,13,14].

Analytical ultracentrifugation measurements show that Arap3-Sam is a monomer in solution, in fact the experimentally measured molecular weight (10.84 kDa) is in perfect agreement with the expected molecular weight. Moreover, no concentration dependent changes can be noticed in [^1^H, ^15^N]-HSQC spectra of Arap3-Sam recorded at protein concentrations of 150 *μ*M or 1.4 mM.

**Figure 1:**
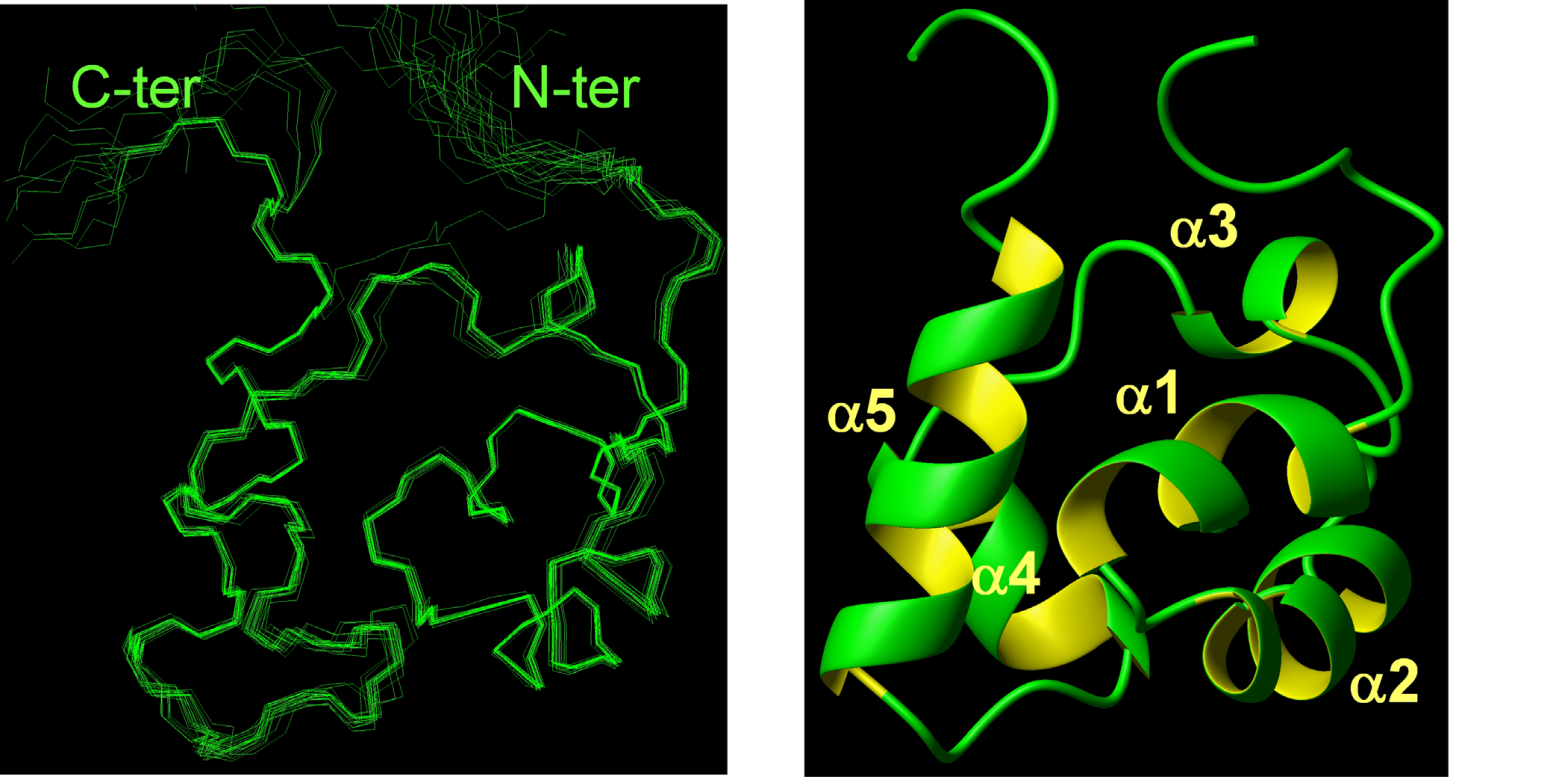
**Solution Structure of Arap3-Sam**. *Left panel*. Superposition on the backbone atoms (residues 27-84) of the 20 NMR structures of Arap3-Sam. The flexible C-terminal tail (residues 88-100) has been omitted for clarity. The final structure calculation includes 1127 upper distance limits (382 intra-residue, 215 short-range, 252 medium-range, 278 long-range), 352 angle constraints and stereospecific assignments for the methyl groups of L33, L38, L50, L59, L64, L67, L79, L81, L82 and V36. *Right panel*. Ribbon drawing of the Arap3-Sam NMR conformer with the lowest CYANA target function. It presents the following secondary structure elements: α 1 (residues 29-36), α 2 (residues 39-47), α 3 (residues 53-56), α 4 (residues 61-67), α 5 (residues 72-82).

The solution structure of Arap3-Sam corresponds to a canonical Sam domain fold (Figure 1, right panel). Relevant structural parameters are reported in Table 1. Distance and angle constraints are well satisfied in the ensemble of structures (Table 1), and the conformers converge well (Figure 1, left panel) as demonstrated by the low root-mean-square deviation (rmsd) values evaluated for the residues of the core domain (Table 1).

**Table 1:** Statistics for the NMR ensemble of Arap3-Sam

NOE upper distance limits	1127
Angle constraints	352

Residual target function, Å^2^	1.16 ± 0.15

Residual NOE violations	

Number > 0.1 Å^#^	3

Residual angle violations	

Number, >5° ^#^	0

Atomic pairwise RMSD, Å	

Backbone atoms (aa 27-84)	0.26 ± 0.10

Heavy atoms (aa 27-84)	0.75 ± 0.09

Ramachandran analysis ^@^	

Residues in core regions	87.2%

Residues in allowed regions	12.2%

Residues in generous regions	0.0%

Residues in disallowed regions	0.6%

^# ^Mean CYANA [30] violations

^@ ^PROCHECK_NMR [34] statistics for residues 27-87

### Ship2-Sam/Arap3-Sam Binding Studies

**Figure 2:**
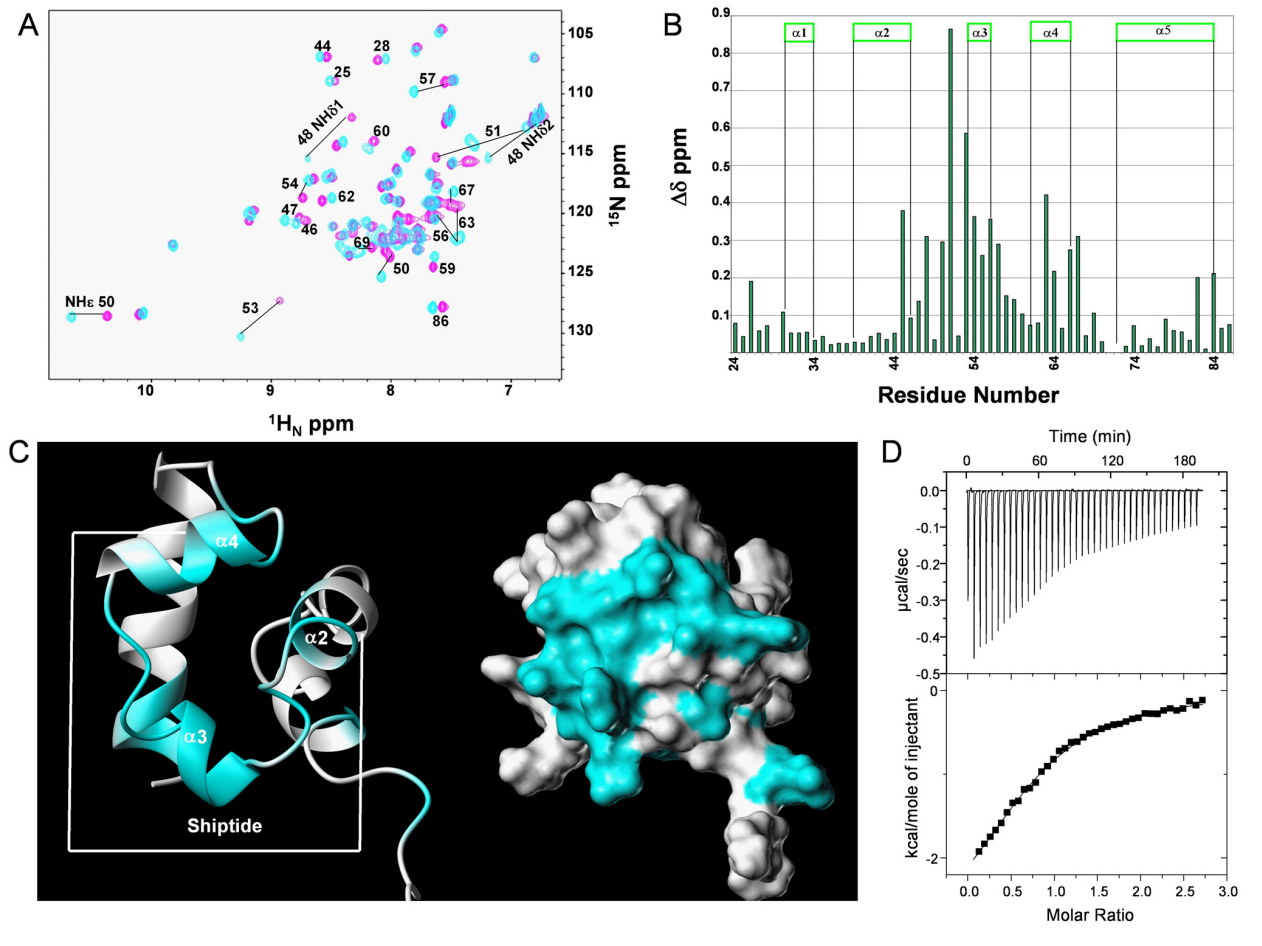
**Mapping the Ship2-Sam binding interface for Arap3-Sam**. *A*. Overlay of [^1^H, ^15^N]-HSQC spectra of ^15^N labeled Ship2-Sam (200 μM) in absence (magenta) and presence (cyan) of unlabeled Arap3-Sam (233 μM). *B*. Graph reporting the normalized chemical shift deviations (Δδ = [(ΔH_N_)^2 ^+ (0.17 * Δ^15^N)^2^]^1/2^) *versus *the residue number. Residues E26, S30, L45, H47, N48, W50, D51, L53, E54, F55, L56, S57, D58, I59, T60, D63, L64, E66, A67, V69, L82, L84 show normalized deviations with values higher that 0.1 ppm. Residues 29 and 71 have not been reported due to spectral overlaps. *C*. Amino acids with normalized chemical shifts deviations (Δδ values) greater than 0.1 ppm are colored in cyan on the 3D solution structure of Ship2-Sam (conformer number 1, pdb code: 2K4P[10]) in its ribbon (left panel) and surface (right panel) representations. The peptide region corresponding to the Shiptide is underlined in the left panel.* D*. ITC data showing the Arap3-Sam (75 μM) titration with the Shiptide peptide (1 mM). The solid line in the lower panel represents the fit of the calorimetric data to a single binding site model.

The interaction between Ship2-Sam and Arap3-Sam was analyzed by means of chemical shift perturbation studies with 2D [^1^H, ^15^N]-HSQC experiments [15,16]. First, to map the Ship2-Sam binding interface for Arap3-Sam, 2D [^1^H, ^15^N]-HSQC spectra of a ^15^N uniformly labeled Ship2-Sam sample were acquired in presence and absence of unlabeled Arap3-Sam (Figure 2A). Normalized chemical shift variations were then evaluated with the equation Δ*δ *= [(ΔH_N_)^2 ^+ (0.17 × Δ^15^N)^2^]^1/2 ^(Figure 2B) [17]. The largest Δ*δ *(values > 0.1 ppm) were mainly found in the middle part of the protein, including the α 3 and α 4 helices, the C-terminal portion of α 2 helix, and the close loop regions (Figure 2B, C).

**Figure 3:**
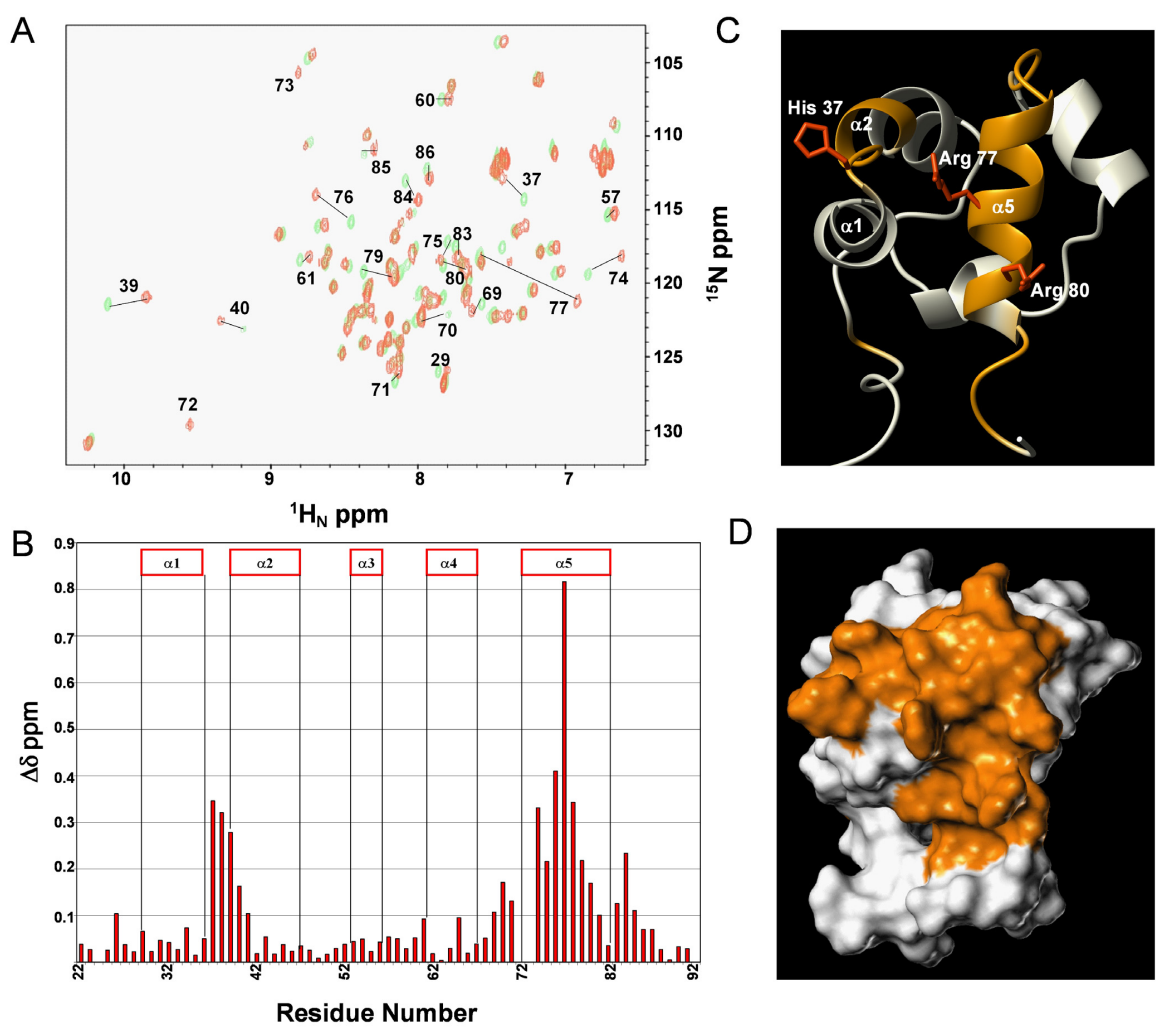
**Mapping the Arap3-Sam binding interface for Ship2-Sam**.* A*. Superposition of [^1^H, ^15^N]-HSQC spectra of ^15^N labeled Arap3-Sam (100 μM concentration) in the apo form (green) and after addition of unlabeled Ship2-Sam (200 μM) (orange). *B*. Histogram of normalized chemical shift deviations (Δδ = [(ΔH_N_)^2 ^+ (0.17 * Δ^15^N)^2^]^1/2^) as function of the residue number. Δδ values > 0.1 ppm are observed for residues D26, H37, L38, E39, Q40, Y41, I69, S70, A71, H74, R75, K76, R77, I78, L79, R80, L81, Q83, T84, G85. Residues T72 and G73 have been omitted from the graph since the corresponding peaks can only be seen in the spectrum of the bound form. *C*. Ribbon drawing of Arap3-Sam (conformer number 1 of the NMR ensemble, pdb code: 2KG5) where the residues with the largest normalized chemical shift deviations (Δδ values > 0.1 ppm) are colored in orange; the side chains of the residues that we mutated, are also shown. *D*. Surface representation of Arap3-Sam in the same orientation and colored as in panel C.

To identify the binding interface of Arap3-Sam for Ship2-Sam, titration experiments were carried out with ^15^N labeled Arap3-Sam and unlabeled Ship2-Sam (Figure 3A). The residues of Arap3 that were greatly perturbed by the interaction were assessed by analyzing the normalized chemical shift deviations (Figure 3B). The largest variations were observed at the α 5 helix and the adjacent α1α2 and α 4α 5 loop regions (Figure 3B, C, D).

The chemical shift mapping results were further supported by isothermal titration calorimetry studies with the Ship2-peptide Ac-EGLVHNGWDDLEFLSDITEEDL-NH2 (Shiptide) that we have previously identified [10]. This peptide encompasses a region of Ship2-Sam (amino acids from 43 to 64) highly affected by chemical shifts variations upon binding to Arap3-Sam (Figure 2C, left panel). ITC measurements proved that the Shiptide could interact with Arap3-Sam with a dissociation constant K_d _of 40 ± 7 μM, a single binding site model (binding stoichiometry n = 0.8 ± 0.1), binding enthalpy Δ*H* = -2602 ± 922 cal/mol and entropy change Δ*S* = 11 ± 3 cal/(mol K) (Figure 2D and Additional File 1).

**Figure 4:**
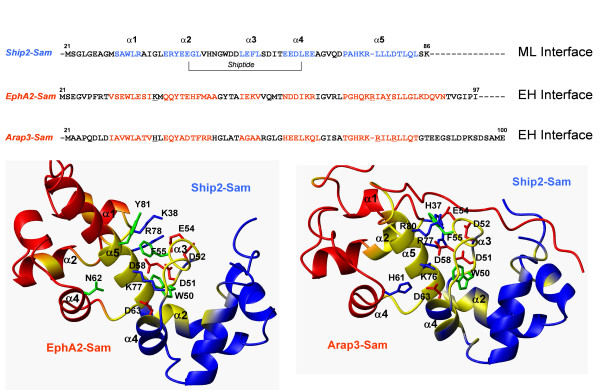
**Docking Studies**. *Upper panel*. Sequence alignment, generated with ClustalW2 [36] of Ship2-Sam, EphA2-Sam and Arap3-Sam. Residues in the helices are colored in blue on the primary sequence of Ship2-Sam and red on the primary sequences of EphA2-Sam and Arap3-Sam. The Shiptide region is indicated. Underlined residues on the EphA2-Sam and Arap3-Sam sequences are the amino acids used in mutagenesis studies (See also [10]). *Lower panel*. Comparison between the best scoring Haddock [18] models of the EphA2-Sam/Ship2-Sam (Haddock score: -81.76) [10] (left) and Arap3-Sam/Ship2-Sam complexes (Haddock score: -150.72) (right). Residues which are most affected by binding in our chemical shifts perturbation studies (Δδ > 0.1 ppm) have been colored in yellow on the ribbon representations of the molecules. The side chains of the residues that may contribute to important interactions at the dimer interface are shown as neon representation.

Molecular modelling studies were also performed with the software Haddock 1.3 [18] to generate a model of the Ship2-Sam/Arap3-Sam complex (see Materials and Methods section for details on the docking procedure). The best Haddock solution (i.e., the one with the lowest Haddock score) is shown in Figure 4. The dimer interface is mainly stabilized by electrostatic interactions in between acidic residues of Ship2-Sam and basic residues of Arap3-Sam (Figure 4). This model has been further confirmed by mutagenesis studies. To this end, an Arap3-Sam triple mutant was designed with the positively charged residues H37, R77 and R80 replaced by negatively charged Asp residues. This mutant fails to bind Ship2-Sam with high affinity as evaluated by chemical shift perturbation studies (See Additional File 2).

### NMR displacement experiment

To assess if Arap3-Sam could compete with EphA2-Sam for the same binding site of Ship2-Sam, we performed 2D [^1^H, ^15^N]-HSQC displacement experiments [19] (See Additional File 3). We first recorded a spectrum of ^15^N labeled EphA2-Sam in its apo form. Then, we added to this sample unlabeled Ship2-Sam in order to observe changes in the 2D [^1^H, ^15^N]-HSQC indicative of the formation of the Ship2-Sam/EphA2-Sam complex. The spectrum of unbound EphA2-Sam could be restored by addition of unlabeled Arap3-Sam (final ratio EphA2-Sam/Ship2-Sam/Arap3-Sam equal to 1:1.8:3.2) (Additional File 3), thus demonstrating dissociation of the Ship2-Sam/EphA2-Sam complex due to the capture of Ship2-Sam by Arap3-Sam.

## Discussion

Sam domains are small protein modules that act in several biological events and can form homotypic or heterotypic associations [20,21]. The lipid phosphatase Ship2 [5,6] contains a Sam domain at its C-terminus. Binding partners of this Sam domain have been only recently reported and consist of the Sam domain from the EphA2 receptor [11] and the Sam domain from the PI3K effector protein Arap3 [3]. The NMR solution structure of Ship2-Sam (pdb code: 2K4P) has been recently determined in our laboratory [10]. We have also investigated the interaction between Ship2-Sam and EphA2-Sam by means of ITC and NMR chemical shift perturbation studies [10]. ITC data have shown that the Sam domain of Ship2 binds to the Sam domain of the EphA2 receptor with a dissociation constant K_d _= 0.75 ± 0.12 μM and a 1:1 binding stoichoimetry [10].

To gain additional insights into the structural determinants characteristic of the Sam-Sam interactions which involve Ship2, we have now performed similar structural and binding studies on the association of Ship2-Sam with Arap3-Sam. ITC data by Ramajjmaker et al. [3] have already reported a K_d _value of about 100 nM and a single binding site model for this interaction.

The solution structure of Arap3-Sam consists of a small five helix bundle and represents a classical Sam-domain fold (Figure 1). In fact, Arap3-Sam presents highest sequence homology with the Sam domain of Arap2 (pdb code: 1X40, Riken Structural Genomics Initiative) as shown by a blastp [22] search of the Arap3-Sam primary sequence *versus *the Protein Data Bank (pdb) database [23]. Sequence similarities with Ship2-Sam (pdb code: 2K4P[10]) and EphA2-Sam (pdb code: 2E8N, Riken Structural Genomics Initiative) are also relatively high (49% and 58% respectively) (Figure 4, upper panel). Previous studies have already reported that like Ship2-Sam, Arap3-Sam does not have a strong propensity to self-associate and prefers to be involved in heterotypic interactions [3]. Our ^15^N R1 and R2 relaxation measurements together with analytical ultracentrifugation studies, further validate these findings.

Chemical shift mapping studies indicate that the interaction surface of Ship2-Sam for Arap3-Sam is mainly made up of the central regions of the protein (Figure 2). This is the same area that we identified as responsible for the binding of Ship2-Sam to EphA2-Sam (Figure 4). The Shiptide, a 22 residue long Ship2-Sam peptide, representing the minimal Ship2-Sam region capable of binding to EphA2-Sam, retains some ability to bind Arap3-Sam as shown by ITC (Figure 2D). In the case of Arap3-Sam/Shiptide interaction, enthalpic contributions are responsible for ~44% of the free energy of binding (Δ*H *= -2.6 kcal/mol, Δ*G *= -5.9 kcal/mol) whereas for EphA2-Sam/Shiptide interaction [10] the enthalpy contributes only ~22% to the free energy of binding (Δ*H *= -1.4 kcal/mol, Δ*G *= -6.5 kcal/mol). Thus, in the Arap3-Sam/Shiptide complex hydrogen bonding and electrostatic interactions are more predominant.

In addition, NMR displacement experiments clearly show that Arap3-Sam and EphA2-Sam compete for the same binding site on the surface of Ship2-Sam (Additional File 3).

The binding area of Arap3-Sam for Ship2-Sam is primarily made up of the C-terminal α 5 helix and adjacent loop regions (Figure 3). Again, the location of this binding site closely resembles the interaction surface of EphA2-Sam for Ship2-Sam. From these binding data, we conclude that Ship2-Sam and Arap3-Sam most likely interact by using the Mid-Loop (ML)/End-Helix (EH) Model that is common among Sam-Sam associations [8,24,25] and where Ship2-Sam and Arap3-Sam are providing the Mid-Loop and End-Helix interfaces respectively (Figure 4). The same interaction mode has been previously proposed by us for the interaction between Ship2-Sam and EphA2-Sam.

A model of the Ship2-Sam/Arap3-Sam complex was generated with the software Haddock 1.3 [18] by using chemical shift perturbation data (Figure 4). The best scoring solution represents well the ML/EH topology that is present in other experimental structure of Sam-Sam complexes [8,25].

The binding site of Ship2-Sam for Arap3-Sam contains many negatively charged residues, while the EH interface of Arap3-Sam includes several positively charged amino acids. As a consequence, this model appears largely stabilized by electrostatic interactions (Figure 4). In fact, by destroying some of these interactions through simultaneous mutation of the positively charged Arap3-Sam residues H37, R77 and R80 to aspartic acids, the binding to Ship2-Sam is abolished or at least highly attenuated as shown by NMR binding data (Additional File 2).

A very similar interaction model has been obtained by us for the Ship2-Sam/EphA2-Sam association, by means of docking procedures [10] (Figure 4). The pattern of interactions at the dimer interface is analogous in the two complexes (Figure 4). It is worth noting that in the Ship2-Sam/EphA2-Sam model the EphA2-Sam residue Y81 may form a stacking π-π interaction with the Ship2-Sam residue F55 that can be replaced by the cation-π interaction between the Arap3-Sam residue R80 and F55 of Ship2 in the complex Ship2-Sam/Arap3-Sam. Furthermore, in the Ship2-Sam/Arap3-Sam complex H61 of Arap3-Sam could provide an additional electrostatic interaction with D63 of Ship2-Sam that is not permitted in the Ship2-Sam/EphA2-Sam dimer (Figure 4). These observations may reflect the relatively stronger binding observed between Arap3-Sam and Ship2-Sam (K_d _= ~0.1 μM [3]) compared to the binding of EphA2-Sam to Ship2-Sam (K_d _= 0.75 ± 0.12 μM) as well as the better Haddock score for the Arap3-Sam/Ship2-Sam model (Figure 4, lower panel).

## Conclusion

We have described the 3D solution structure of Arap3-Sam and reported on binding studies with Ship2-Sam. Our work leads us to hypothesize that the interaction mode of these two Sam domains is best described by the canonical Mid-Loop/End-Helix model [8,24-26] in which Ship2-Sam and Arap3-Sam are providing the Mid-Loop and End-Helix interfaces respectively. A similar model has been recently suggested by us for the interaction in between Ship2-Sam and EphA2-Sam [10]. Our studies also show that Arap3-Sam competes with EphA2-Sam for binding to Ship2-Sam. Together with binding studies on the Shiptide peptide, our results provide a framework onto which one could envision designing novel molecular probes able to selectively interfere with either the Ship2-Sam/EphA2-Sam or with the Ship2-Sam/Arap3-Sam associations.

## Methods

### Protein expression

Ship2-Sam and EphA2-Sam were expressed as previously reported [10]. A synthetic genes construct containing residues from 1 to 80 of human Arap3 (UniprotKB/TrEMBL code: Q8WWN8), encompassing the Sam domain (residues from 4 to 68), was purchased from Celtek (Nashville, TN). Genes were cloned into the PET15b plasmid and transformed using BL21-Gold (DE3) competent cells (Stratagene).

The PET15b plasmid carrying genes for the Arap3-Sam triple mutant (H37D, R77D, R80D) was purchased from Celtek (Nashville, TN). These protein constructs have each an N-terminal 6His-tag (See Additional File 4).

Unlabeled proteins were expressed at 37°C in LB medium. Protein over-expression was induced at OD_600 _= 0.6 for 4 hours with isopropyl β-D-thiogalactopyranoside (IPTG) at 1 mM concentration. Expression of ^15^N/^13^C double labeled and ^15^N labeled proteins was carried out in M9 minimal medium containing 2 g/l of ^13^C-Glucose and/or 0.5 g/l of ^15^NH_4_Cl. 10% fractional ^13^C labeling for stereo-specific assignments of Leu-CH_3_^δ1,2^/Val-CH_3_^γ1,2 ^methyl groups [27] was obtained by adding 3.6 g of ^12^C-glucose (natural abundance) and 0.4 g of ^13^C-glucose to the M9 medium.

After dissolving the pellet in the following buffer: 50 mM Tris (pH = 8), 500 mM NaCl, 5 mM imidazole, cell were harvested by sonication. The protein was purified on a nickel column (His-trap TM FF, 5 ml, Amersham) by using an AKTA prime plus FPLC system; the elution buffer consisted of 50 mM Tris (pH = 8), 500 mM NaCl, 200 mM imidazole. To avoid non-specific interactions with the Shiptide during ITC experiments, the His-tag tail of Arap3-Sam was cut away from the protein by incubating it overnight at 4°C with thrombin. The thrombin was then removed with a benzamidine column (FF (HS), 1 ml, Amersham).

The protein concentration was estimated by a nanodrop ND-1000 spectrometer.

### Resonance assignments of Arap3-Sam

Experiments for resonance assignments were recorded at 25°C on a Bruker Avance 600 MHz or a 700 MHz Bruker AvanceIII spectrometers both equipped with TCI cryoprobes. NMR samples consisted of ^15^N or ^15^N/^13^C labeled Sam domains (1 mM) in phosphate buffer saline (PBS, 11.9 mM phosphate, 137 mM NaCl, 2.7 mM KCl) (Fisher Scientific) at pH = 7.7 with 0.3% NaN_3_. Sample volumes of 500 μl (95% H_2_O/5% D_2_O) were used. The Bruker software Topspin version 2.0 was implemented to process NMR spectra; NEASY [28] was used to analyze the data.

Backbone assignments were obtained by analyzing triple resonance experiments (HNCA, HNCACB, HNCO) [29]. The (H)CC(CO)NH spectrum was used to assign carbon side chains. Comparison of 3D ^15^N resolved-[^1^H, ^1^H] NOESY (100 ms mixing time) and 3D ^15^N resolved-[^1^H, ^1^H] TOCSY (70 ms mixing time) together with analysis of HCCH-TOCSY experiments allowed to assign proton side chains.

H_N_, N_H _and Cα backbone atoms of the Ship2-Sam/Arap3-Sam complex were identified in HNCA experiments acquired on samples containing either ^15^N/^13^C double labeled Ship2-Sam at a concentration of 1 mM and unlabeled Arap3-Sam at a concentration of 1 mM or double labeled Arap3-Sam (1 mM concentration) and unlabeled Ship2-Sam (1 mM concentration). Stereo-specific assignments for Leu-CH_3_^δ ^1,2 and Val-CH_3_^γ ^1,2 methyl groups of Arap3-Sam were obtained by recording a 2D [^1^H, ^13^C]-HSQC experiment of a fractionally ^13^C labeled Arap3-Sam sample at a concentration of 1 mM [27].

### Relaxation measurements

Experiments for measuring backbone ^15^N nuclear spin relaxation parameters, were recorded at 25°C on a 600 MHz Bruker Avance DRX spectrometer equipped with a TXI probe. Longitudinal relaxation rates (R1) and transverse relaxation rates (R2) were obtained for ^15^N-labeled samples of Arap3-Sam at the concentrations of 150 μM and 1.4 mM and for a sample of the Sam-Sam complex containing ^15^N-labeled Ship2-Sam (1 mM) and unlabeled Arap3-Sam (2 mM). R1 and R2 relaxation data were collected and analyzed as we have previously reported for the Ship2-Sam and EphA2-Sam interaction [10]. Briefly, five relaxation delays (0.01, 0.1, 0.3, 0.6, 1.0 s) were used for R1 measurements; and seven relaxation delays were implemented for R2 data sets (i.e., 0.01, 0.03, 0.05, 0.07, 0.11, 0.15, 0.19 s). The rotational correlation time was estimated with the software tmest (Palmer A. G. III, Columbia University) by using the average R2/R1 values.

### Solution structure of Arap3-Sam

Structure calculations were performed with the program CYANA version 2.1 [30]. A 3D-^15^N resolved [^1^H, ^1^H] NOESY-HSQC spectrum [31] (100 ms mixing time) together with a 3D ^13^C resolved [^1^H, ^1^H] NOESY-HSQC spectrum (150 ms mixing time) and 2D [^1^H, ^1^H] NOESY [32] (100 ms mixing time), for the aliphatic to aromatic region, that was recorded after dissolving the lyophilized protein sample in 99% D_2_O, were used to obtain distance constraints. Structure calculations were initiated from 100 random conformers; the 20 structures with the lowest CYANA target functions were analyzed with the programs MOLMOL [33] and PROCHECK-NMR [34]. Colour figures were produced with MOLMOL [33]. MOLCAD [35], as implemented in Sybyl, was used to generate surface representations. NMR structures have been deposited in the Protein Data Bank under accession code 2KG5.

### NMR Binding studies

Chemical shifts perturbation studies by means of 2D [^1^H, ^15^N]-HSQC were carried out to study the protein-protein interaction [15,16]. First, ^15^N-labeled Ship2-Sam (200 μM concentration) was titrated with increasing amounts of unlabeled Arap3-Sam (50, 74, 130, 186, 233 μM). Then, a ^15^N labeled Arap3-Sam sample (200 μM concentration) was titrated with unlabeled Ship2-Sam (concentrations: 50, 100, 200 μM). Details on the NMR chemical shift perturbation studies with the Arap3-Sam triple mutant are reported in the Additional File 2.

2D spectra were compared with the program Sparky (T. D. Goddard and D. G. Kneller, SPARKY 3, University of California, San Francisco).

### Isothermal titration calorimetry

ITC measurements were carried out at 25°C with a VP-ITC apparatus (Microcal, USA). The Shiptide (Ac-EGLVHNGWDDLEFLSDITEEDL-NH_2_) [10], was purchased by the Protein/DNA Facility of the Medical College of Wisconsin. A solution of peptide (1 mM concentration) was titrated into a solution of Arap3-Sam at a concentration of 75 μM. For these studies, the peptide was dissolved in 1 × PBS at pH = 7.7 and Arap3-Sam was extensively dialyzed in the same buffer.

ITC experiments were repeated twice to evaluate the reproducibility of the data. Data were fit to a standard one binding site model using Origin as supplied by Microcal.

### Analytical ultracentrifugation

A Beckman ProteomeLab™ Optima XL-I analytical ultracentrifuge was used to carry out sedimentation equilibrium analysis. Three runs were performed by using samples with protein concentrations of 1 mg/ml, 0.33 mg/ml, and 0.11 mg/ml, respectively. Data were collected at the angular velocity of 30,000 rpm and at 20°C. Data were analyzed with the software HeteroAnalysis (James L. Cole; ).

### Docking studies

The program Haddock 1.3 [18] was used to generate a model of the Ship2-Sam/Arap3-Sam complex. The NMR structures number one of both Ship2-Sam (pdb code: 2K4P, [10]) and Arap3-Sam (pdb code: 2KG5) were implemented for these studies. Chemical shift perturbation data were exploited to produce ambiguous interaction restraints (AIR). Residues H47, N48, W50, D51, D52, E54, F55, S57, D58, I59, T60, E61, E62, E66, Q70 of Ship2-Sam were set as active. For Arap3-Sam, residues H37, S70, T72, G73, K76, R77, R80, Q83 were considered active, whereas H61, E62, E63, K65, Q66 were set as passive. Residues for the AIR restraints were chosen among the ones with the greatest chemical shift perturbation because they either show high solvent exposure (> 30% as evaluated with MOLMOL [33]) or because they could provide interactions at the interface as shown in experimental structures of Sam-Sam complexes. The limit for the AIR restraints was kept to the default value of 2 Å. During the rigid body energy minimization stage, 2000 structures were calculated; in the second iteration a semi-flexible simulated annealing of the best 200 solutions was performed, finally a refinement in water was carried out. Segments 48-66 and 70-80 of Ship2-Sam and Arap3-Sam respectively, were set as semi-flexible and movements of their side-chains were permitted during the semi-rigid body docking protocol. Besides, residues of the C-terminal tail of Arap3-Sam (88-100) were set completely flexible during the whole docking calculation.

## Abbreviations

Arap3, Arf GAP, Rho GAP: Ankyrin repeat and PH domain; Arap3-Sam: Sam domain of Arap3; EH: End-Helix; EphA2-Sam: Sam domain of the EphA2 receptor; FPLC: Fast Performance Liquid Chromatography; GAP: GTP-ase activator protein; HSQC: Heteronuclear Single Quantum Coherence Spectroscopy; IPTG: isopropyl β-D-thiogalactopyranoside; ITC: isothermal titration calorimetry; MD: Mid-Loop; NOESY: Nuclear Overhauser Enhancement Spectroscopy; PBS: phosphate buffer saline; PDK1: 3-phosphoinositide-dependent protein kinase-1; PI3K: phospatidylinositol 3 kinase; PtIns(3,4,5)P3: phosphatidylinositol(3,4,5)P3; rmsd: root-mean-square deviation; Sam: Sterile Alpha Motif; Ship2: Src homology 2 domain-containing phosphoinositide-5-phosphatase 2; Ship2-Sam: Sam domain of Ship2; TOCSY: Total Correlation Spectroscopy.

## Authors' contributions

ML and MP designed the study; ML performed the research; JC performed ITC studies; ML and MP wrote the manuscript. All authors read and approved the final manuscript.

## Supplementary Material

Additional file 1 **ITC control experiment**. Calorimetric curve showing the titration of the Shiptide *versus *buffer. Click here for file

Additional file 2 **Chemical shift perturbation studies with the Arap3-Sam mutant**. The comparison of 2D [^1^H, ^15^N]-HSQC spectra of ^15^N labeled Arap3-Sam mutant in absence and presence of unlabeled Ship2-Sam and the overlay of 2D [^1^H, ^15^N]-HSQC spectra of ^15^N labeled Ship2-Sam in absence and presence of unlabeled Arap3-Sam mutant, are reported. Click here for file

Additional file 3 **Displacement experiment**. The 2D [^1^H, ^15^N]-HSQC spectra of ^15^N labeled EphA2-Sam in its apo form, bound to Ship2-Sam and in presence of both Ship2-Sam and Arap3-Sam, are shown. Click here for file

Additional file 4 **Protein sequences**. The amino acid sequence of the wild-type and mutant proteins, we used in this study, are here listed. Click here for file
