# Supplementary material for: The Sam domain of the lipid phosphatase Ship2 adopts a common model to interact with Arap3-Sam and EphA2-Sam
Source: BMC Struct Biol. 2009 Sep 18;9:59. doi: 10.1186/1472-6807-9-59 (PMC2755476; doi:10.1186/1472-6807-9-59)
Supplement: Additional file 1 — ITC control experiment. Calorimetric curve showing the titration of the Shiptide versus buffer. [file 1472-6807-9-59-S1.DOC]

**ITC control experiment**. Calorimetric curve showing the titration of the Shiptide against the buffer (1X PBS pH=7.7).
