# Supplementary material for: The Sam domain of the lipid phosphatase Ship2 adopts a common model to interact with Arap3-Sam and EphA2-Sam
Source: BMC Struct Biol. 2009 Sep 18;9:59. doi: 10.1186/1472-6807-9-59 (PMC2755476; doi:10.1186/1472-6807-9-59)
Supplement: Additional file 2 — Chemical shift perturbation studies with the Arap3-Sam mutant. The comparison of 2D [1H, 15N]-HSQC spectra of 15N labeled Arap3-Sam mutant in absence and presence of unlabeled Ship2-Sam and the overlay of 2D [1H, 15N]-HSQC spectra of 15N labeled Ship2-Sam in absence and presence of unlabeled Arap3-Sam mutant, are reported. [file 1472-6807-9-59-S2.DOC]

**Chemical shift perturbation studies with the Arap3-Sam triple mutant (H37D, R77D, R80D).** (Left panel) Superposition of 2D [^1^H, ^15^N]-HSQC spectra of ^15^N labeled Arap3-Sam mutant (150 μM) in absence (cyan) and presence (magenta) of unlabeled Ship2-Sam (1 mM). (Right panel) Comparison of 2D [^1^H, ^15^N]-HSQC spectra of ^15^N labeled Ship2-Sam (70 μM) in absence (cyan) and presence (magenta) of unlabeled Arap3-Sam mutant (400 μM). These chemical shift perturbation studies clearly indicate the absence of a strong affinity interaction in between the triple mutant Arap3-Sam and Ship2-Sam. In fact, major differences between spectra recorded in presence and absence of an excess of ligand cannot be observed. Very small changes in the spectra are more likely caused by dilution effects.

Due to the very low affinity of the interaction and lower stability of the mutant protein, we are unable to get reliable ITC data and determine a k_d_ for the binding of the mutant Arap3-Sam to Ship2-Sam.
