# Supplementary material for: The Sam domain of the lipid phosphatase Ship2 adopts a common model to interact with Arap3-Sam and EphA2-Sam
Source: BMC Struct Biol. 2009 Sep 18;9:59. doi: 10.1186/1472-6807-9-59 (PMC2755476; doi:10.1186/1472-6807-9-59)
Supplement: Additional file 3 — Displacement experiment. The 2D [1H, 15N]-HSQC spectra of 15N labeled EphA2-Sam in its apo form, bound to Ship2-Sam and in presence of both Ship2-Sam and Arap3-Sam, are shown. [file 1472-6807-9-59-S3.DOC]

**Displacement experiment.** (Left panel) Superposition of 2D [^1^H, ^15^N]-HSQC spectra of a ^15^N labeled EphA2-Sam sample (120 μM) in absence (red) and in presence of Ship2-Sam (218 μM) (blue). (Right panel) Overlay of 2D [^1^H, ^15^N]-HSQC spectra of EphA2-Sam in the unbound form (red) and in presence of both Ship2-Sam (218 μM) and Arap3-Sam (389 μM) (green). Due to dilution effects, the spectrum in green has been recorded by doubling the number of scans with respect to the other spectra.
