# Supplementary material for: The Sam domain of the lipid phosphatase Ship2 adopts a common model to interact with Arap3-Sam and EphA2-Sam
Source: BMC Struct Biol. 2009 Sep 18;9:59. doi: 10.1186/1472-6807-9-59 (PMC2755476; doi:10.1186/1472-6807-9-59)
Supplement: Additional file 4 — Protein sequences. The amino acid sequence of the wild-type and mutant proteins, we used in this study, are here listed. [file 1472-6807-9-59-S4.DOC]

Arap3-Sam sequences

*Arap3-Sam wild-type protein*

MGSSHHHHHHSSGLVPRGSHMAAPQDLDIAVWLATVHLEQYADTFRRHGLATAGAARGLGHEELKQLGISATGHRKRILRLLQTGTEEGSLDPKSDSAME

*Arap3-Sam triple mutant protein (H37D, R77D, R80D)*

MGSSHHHHHHSSGLVPRGSHMAAPQDLDIAVWLATVDLEQYADTFRRHGLATAGAARGLGHEELKQLGISATGHRKDILDLLQTGTEEGSLDPKSDSAME
